# Além da Complexidade da Lesão: Precisamos de um Novo Escore Para Oclusões Coronárias Crônicas?

**DOI:** 10.36660/abc.20260506

**Published:** 2026-08-03

**Authors:** Pedro Beraldo de Andrade, Thyago Furquim

**Affiliations:** 1 Santa Casa de Misericórdia de Marília Marilia SP Brasil Santa Casa de Misericórdia de Marília, Marilia, SP – Brasil; 2 UNICAMP Campinas SP Brasil Universidade Estadual deCampinas (UNICAMP), Campinas, SP – Brasil

**Keywords:** Oclusão coronária, Circulação colateral, Inflamação

O escore HALP (Hemoglobina, Albumina, Linfócitos e Plaquetas) é um índice que combina parâmetros laboratoriais facilmente disponíveis e reflete o estado inflamatório e nutricional de um indivíduo. Tradicionalmente utilizado em doenças oncológicas^1^ e inflamatórias,^2^ recentemente emergiu como um marcador prognóstico em diversas condições cardiovasculares, incluindo síndromes coronárias agudas,^3^ insuficiência cardíaca^4^ e fibrilação atrial não valvar.^5^ Valores de HALP mais baixos têm sido associados a um estado pró-inflamatório, homeostase hematológica prejudicada e pior estado nutricional.

Esses fatores podem afetar negativamente os processos de remodelação vascular, levando à angiogênese patológica.^6^ Nesse contexto, Akkaya e Öztürk analisaram retrospectivamente 269 pacientes com oclusões coronárias crônicas identificada por angiografia coronária e demonstraram que o escore HALP apresentou excelente desempenho discriminatório para a detecção de circulação colateral bem desenvolvida.^7^ Conforme relatado nesta edição dos Arquivos Brasileiros de Cardiologia, o escore HALP foi significativamente maior em pacientes com circulação colateral proeminente em comparação com aqueles com colaterais pouco desenvolvidas (75,5 ± 13,9 vs. 35,1 ± 11,3; p < 0,001) e foi um preditor independente desse achado angiográfico (OR: 0,88; IC 95%: 0,83–0,93; p < 0,001). A análise da curva ROC (*Receiver Operating Characteristic*) demonstrou uma AUC de 0,958 (IC 95%: 0,93–0,98), com um valor de corte >49,08, resultando em uma sensibilidade de 91,3% e uma especificidade de 90,8%. Em um estudo semelhante, Süleymanoğlu et al. avaliaram retrospectivamente 240 pacientes com oclusão coronária crônica e confirmaram que o escore HALP estava positivamente associado à qualidade da circulação colateral, avaliada pela classificação de Werner, permanecendo um forte preditor independente na análise multivariável.^8^

A indicação mais robusta para o tratamento de oclusões coronárias crônicas continua sendo o alívio dos sintomas de angina.^9^ Uma vez selecionada a intervenção coronária percutânea, o planejamento do procedimento se baseia principalmente na angiografia invasiva, cada vez mais complementada pela angiotomografia coronária, que permite a identificação de canais colaterais intervencionáveis. Diversos sistemas de escore angiográfico, incluindo J-CTO (*Multicenter CTO Registry in Japan*), PROGRESS-CTO (*Prospective Global Registry of Chronic Total Occlusion Interventions*), CASTLE [idade na cirurgia de revascularização do miocárdio (≥70 anos), anatomia do coto (obtuso ou invisível), grau de tortuosidade (grave ou não visível), comprimento da oclusão (≥20 mm) e extensão da calcificação (grave)] e CT-RECTOR (*Computed Tomography Registry of Chronic Total Occlusion Revascularization*), auxiliam na predição do sucesso técnico.^10^ Nesse contexto, precisamos de um novo escore capaz de predizer a probabilidade de identificar canais colaterais adequados para intervenção, a fim de sistematizar ainda mais o manejo de pacientes com oclusões coronárias crônicas? A resposta provavelmente é não, visto que a avaliação anatômica por meio de angiografia invasiva ou tomografia computadorizada coronária é invariavelmente necessária, e tal escore dificilmente influenciaria substancialmente esse processo de tomada de decisão.

Poderia o escore HALP agregar valor clínico prognóstico à tomada de decisão quanto à indicação ou não de revascularização miocárdica em pacientes com oclusão coronária crônica? Trata-se de um conceito potencialmente relevante a ser explorado, uma vez que a qualidade da circulação colateral estaria relacionada a menor extensão de isquemia, melhor preservação da função ventricular esquerda, menor remodelamento ventricular e maior viabilidade miocárdica.^11^ Linhas de pesquisa futuras poderiam investigar o poder preditivo do escore HALP para identificar pacientes que se beneficiariam da estratégia de revascularização, para além da melhora de sintomatologia, comparando o prognóstico de pacientes submetidos a tratamento bem-sucedido de acordo com a categoria de escore HALP alto ou baixo, acrescentando, assim, uma ferramenta adicional à difícil tomada de decisão na condução de pacientes portadores de oclusões coronárias crônicas.
