# Supplementary material for: Além da Complexidade da Lesão: Precisamos de um Novo Escore Para Oclusões Coronárias Crônicas?
Source: Arq Bras Cardiol. 2026 Aug 3;123(7):e20260506. [Article in Portuguese] doi: 10.36660/abc.20260506 (PMC13668444; doi:10.36660/abc.20260506)
Supplement: Translation

# Translation: Beyond Lesion Complexity: Do We Need a New Score for Chronic Total Occlusions?

**DOI:** 10.36660/abc.20260506i

**Authors:** Pedro Beraldo de Andrade, Thyago Furquim

**Affiliations:** 1 Brazil Santa Casa de Misericórdia de Marília, Marilia, SP – Brazil; 2 Brazil Universidade Estadual de Campinas (UNICAMP), Campinas, SP – Brazil

**Keywords:** Coronary Occlusion, Collateral Circulation, Inflammation

The Hemoglobin, Albumin, Lymphocyte, and Platelet (HALP) score is an index that combines readily available laboratory parameters and reflects an individual’s inflammatory and nutritional status. Traditionally used in oncologic^1^ and inflammatory diseases,^2^ it has recently emerged as a prognostic marker in various cardiovascular conditions, including acute coronary syndromes,^3^ heart failure,^4^ and non-valvular atrial fibrillation.^5^ Lower HALP values have been associated with a pro-inflammatory state, impaired hematologic homeostasis, and poorer nutritional status.

These factors may adversely affect vascular remodeling processes, leading to pathological angiogenesis.^6^ In this context, Akkaya and Öztürk retrospectively analyzed 269 patients with chronic total coronary occlusion identified by coronary angiography and demonstrated that the HALP score exhibited excellent discriminatory performance for the detection of well-developed collateral circulation.^7^ As reported in the current issue of the *Arquivos Brasileiros de Cardiologia*, the HALP score was significantly higher in patients with prominent collateral circulation compared with those with poorly developed collaterals (75.5 ± 13.9 vs. 35.1 ± 11.3; p < 0.001), and was an independent predictor of this angiographic finding (OR: 0.88; 95% CI: 0.83–0.93; p < 0.001). Receiver operating characteristic (ROC) curve analysis demonstrated an AUC of 0.958 (95% CI: 0.93–0.98), with a cutoff value >49.08, yielding a sensitivity of 91.3% and a specificity of 90.8%. In a similar study, Süleymanoğlu et al. retrospectively evaluated 240 patients with chronic total coronary occlusion and confirmed that the HALP score was positively associated with collateral circulation quality, as assessed by the Werner classification, remaining a strong independent predictor in multivariable analysis.^8^

The most robust indication for the treatment of chronic total coronary occlusions remains the relief of anginal symptoms.^9^ Once percutaneous coronary intervention is selected, procedural planning is based primarily on invasive angiography, increasingly complemented by coronary computed tomography angiography, which allows identification of interventional collateral channels. Several angiographic scoring systems, including J-CTO (Multicenter CTO Registry in Japan), PROGRESS-CTO (Prospective Global Registry of Chronic Total Occlusion Interventions), CASTLE [CABG age (≥70 years), stump anatomy (blunt or invisible), tortuosity degree (severe or unseen), length of occlusion (≥20 mm), and extent of calcification (severe)], and CT-RECTOR (Computed Tomography Registry of Chronic Total Occlusion Revascularization), assist in predicting technical success.^10^ In this context, do we need a new score capable of predicting the likelihood of identifying collateral channels suitable for intervention in order to further systematize the management of patients with chronic total coronary occlusions? The answer is probably no, since anatomical assessment through invasive angiography or coronary computed tomography is invariably required, and such a score would be unlikely to substantially influence this decision-making process.

Could the HALP score provide additional prognostic value in guiding revascularization decisions for patients with chronic total coronary occlusions? This is a potentially relevant concept worthy of further investigation, as the quality of collateral circulation has been associated with lower ischemic burden, better preservation of left ventricular function, reduced ventricular remodeling, and greater myocardial viability.^1 1^ Future studies could evaluate the predictive value of the HALP score for identifying patients most likely to benefit from a revascularization strategy beyond symptomatic improvement. Comparing outcomes among patients undergoing successful chronic total occlusion recanalization between high- and low-HALP score categories could provide important insights into HALP’s potential role in patient selection, ultimately adding another tool to support decision-making for one of the most challenging aspects of managing chronic total coronary occlusions.
